# Novel Influence Diagnostics in Multistate Models for Breast Cancer

**DOI:** 10.1002/sim.70556

**Published:** 2026-04-20

**Authors:** Valeria Leiva‐Yamaguchi, Alejandra Tapia, Manuel Galea, Oscar M. Rueda

**Affiliations:** ^1^ MRC Biostatistics University of Cambridge Cambridge UK; ^2^ Facultad de Matemáticas Pontificia Universidad Católica de Chile Santiago Chile

**Keywords:** breast cancer, case‐weight perturbation, cox‐partial likelihood function, influence diagnostics, likelihood inference

## Abstract

Multistate models were developed to model survival data where several midpoints and endpoints are of interest; and they have been particular successful in modeling dynamics of cancer. As in any statistical model, identification of influential observations is an essential task, as they can significantly affect the validity of inferred parameters and conclusions drawn from the data. The local influence approach is a set of methods designed to detect the effect of small perturbations of the model or data on the inference, allowing for deeper data analysis. In this paper, we derive local influence methods for multistate models and illustrate their use with a breast cancer dataset. In particular, we develop and implement local influence diagnostic techniques based on a suitable estimation equation. For simplicity, we restrict our consideration to the Multistate Proportional Hazards model, using different case‐weight perturbation strategies.

## 1 Introduction

Breast cancer is the most common oncological disease worldwide in women. Although early detection and available treatments have advanced in recent years, the mortality rate remains high in the population (https://www.who.int/news‐room/fact‐sheets/detail/breast‐cancer). Although the goal of surgery and associated treatments is to kill all cancer cells, some may escape treatment and survive. These undetected cells can multiply and invade other tissues, sometimes months or several years after surgery. These relapses can occur in the vicinity of the primary tumor (locoregional relapse), or in other parts of the body (distant relapse). The prognosis of patients after diagnosis depends on several clinico‐pathological factors, and in the case of metastatic patients, on when and where a recurrence occurs [1]. Moreover, these types of events are related, so traditional survival models that focus on a single endpoint present some limitations: they do not capture recurrence patterns or they only consider death due to a specific cause of the disease as the primary endpoint, censoring deaths from other causes. A class of statistical models known as multistate models has become a useful tool for studying the progression of diseases such as breast cancer, considering the transitions between the different health states of patients. For more details and recent applications of this type of models in medical sciences see [2, 3, 4, 5, 6, 7].

Multistate models track the evolution of a patient's condition over time. This type of analysis considers several states and the main objective is to evaluate the process of moving from one state to another, called a transition. The estimation of transition rates is a fundamental aspect in this type of models, since they provide us with information about the relationship between the different intermediate events or endpoints, for example, the association between local relapse and distant relapse and survival in breast cancer [8], as well as the influence of prognostic factors on each of the transition rates [9]. The hazard ratios in each transition can be modeled through a parametric or semiparametric model [8], and the combination of them allows the prediction of the prognosis of a patient at different stages of the clinical pathway. Multistate models have the ability to make predictions taking into account patient characteristics and clinical factors available at time of prediction, in addition to considering the full trajectory of the patient's history and the times where intermediate events occurred [10].

Considering the importance of identifying the patients who are likely to relapse, and the additional complexities that multistate models add to standard single endpoint survival analysis, it is crucial to conduct a sensitivity analysis of the model to evaluate the results of model fitting and the predictions. This is because multistate models, like any other statistical model, are almost always approximate descriptions of more complicated processes and are therefore almost always imprecise. Moreover, our sample may contain extreme values, atypical of the population, or even errors in data collection or transcription. Due to this inaccuracy, the study of the variation of the results of the analysis under modest modifications of the model or data becomes important. If a minor modification of an approximate description seriously influences key results of a model, there is surely cause for concern. On the other hand, if such modifications are found to be unimportant, the model and our sample are robust with respect to the induced perturbations and our ignorance of the true underlying model will do no harm [11].

Following [12], there are various alternatives for evaluating the influence of perturbations in the data and in the assumptions of the model regarding the parameter estimators of interest. The deletion of cases is a common diagnostic technique for evaluating the effect of an observation in the estimation process. This is an analysis of global influence, since the effect of an observation (or a group of observations) is quantified by eliminating it from the data set. However, this method has two drawbacks that limit its application: (i) it is difficult to apply to statistical models for complex data, due to its high computational cost, and (ii) in statistical models for dependent data it is not possible to isolate the effect of one observation of the phenomenon, see, for instance; References [13, 14]. Alternatively, Reference [11] proposes an interesting method, called local influence, to evaluate the effect of small perturbations in the data and in the assumptions of the statistical model regarding the maximum likelihood estimators, without eliminating the observations. That is, it verifies the existence of observations that under modest modifications in the model or data cause disproportionate variations in the results; see [11]. This technique has been applied to a wide range of models, such as mixed models [15, 16], multivariate regression models with measurement error [17], spatial linear models [18], survival analysis [19, 20], and [21], among many other statistical models.

In this article, we focus primarily on proposing diagnostic measures based on the local influence method in the continuous time Cox Proportional Hazards multistate model under right censoring, and address the case weight perturbation scheme, which is often the basis for the study of local influence; see [11]. Then, since each component of the likelihood has the same weight in the logarithm of the partial likelihood function of the Cox model (model assumption), the purpose of this scheme is to evaluate the sensitivity of maximum likelihood estimators under small modifications in these components. However, different approaches to these components will allow us to propose three perturbation strategies. These are: (i) perturbation of each individual in a specific transition, (ii) perturbation of each transition, and (iii) perturbation of each individual. These three perturbation strategies will allow us to evaluate the assumption of equal weights of the defined components, as well as to identify anomalous components that could affect statistical inference. These strategies will also allow us to obtain valuable information about specific patients, transitions, or both that need to be manually inspected and checked, enabling us to gain a better perspective on the robustness of our model when influential components are detected. Thus, the case‐weight perturbation scheme will help identifying if results are sensitive to variations in contributions, potentially leading to a redefinition of the model; and eventually proposing alternative (and more robust) statistical models.

## 2 Study Cohort and Multistate Model

We model disease progression using a multistate framework, which allows transitions between clinically relevant states such as disease‐free, local relapse, distant relapse, and death. The multistate model provides a flexible structure to analyze the time to each event while accounting for intermediate transitions. Details on the model formulation are provided in Section 2.2.

### 2.1 Breast Cancer Dataset

To illustrate and validate this modeling approach, we applied it to patient‐level data from the Molecular Taxonomy of Breast Cancer International Consortium (METABRIC) [22]. Although the goal of the study was to characterize the genomic and transcriptomic architecture of 1980 breast cancers, here we will analyze a larger dataset containing only clinico‐pathological information from 3240 patients collected between 1977 and 2005 in 5 hospitals in the United Kingdom and Canada, with a median follow‐up of 14 years. Observations are right censored, and full details on the dataset can be found in [1]. Table 1 summarizes the most relevant prognostic factors:

**TABLE 1:** Prognostic factors for all patients (3219).

Prognostic factor	(%)
Tumor size	
≤20mm	1330 (41.9)
20−50mm	1674 (52.7)
>50mm	170 (5.4)
Missing	45
ER status	
Negative	850 (27.0)
Positive	2297 (73.0)
Missing	72
Grade of tumor	
Low grade	253 (8.3)
Intermediate grade	1246 (40.9)
High grade	1551 (50.9)
Missing	169
Nodes positive	
≤4	2847 (88.4)
>4	372 (11.6)
Age (years)	
≤65	1879 (58.4)
>65	1340 (41.6)

Following our previous analysis [1], we eliminated samples that lacked follow‐up time and those classified as stage 4. Additionally, we discarded samples without follow‐up time or known death status. We also excluded benign cases, ductal carcinoma in situ (DCIS), or phyllodes tumors (PHYL). As in the original study, we made some adjustments on the transitions: local relapses that happen after distant relapses were not considered. After removing missing observations, we ended up with *M* = 2951 patients. To further clarify the data structure and facilitate interpretation of the model results, we have included Tables B3 and B4 in the Appendix B. These tables present the frequency of transitions between clinical states (post‐surgery, local relapse, distant relapse, cancer death, natural death) separately for ER− and ER+ patients. They provide both the number of patients entering each state and the number of observed events for each transition, offering a comprehensive overview of the state dynamics within each subgroup.

### 2.2 Multistate Model

**FIGURE 1:**
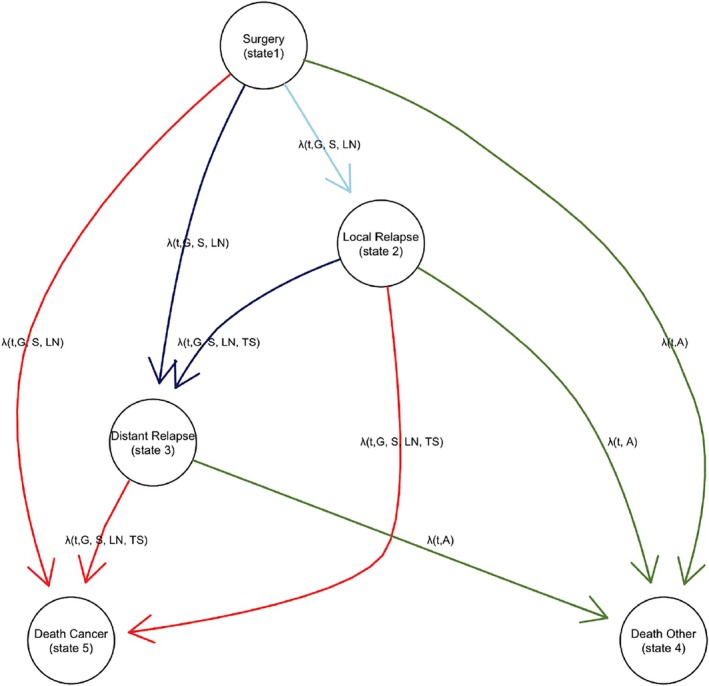
Graphical representation of the model. The model is visually depicted with nodes symbolizing potential states and arcs symbolizing possible transitions between those states. The variables that can impact each transition rate are annotated in each arrow: G = Tumor Grade, S = Tumor Size, A = Age, LN = Number of positive lymph nodes, TS = Time of surgery.

We adopted the multistate model from [1]. Figure 1 illustrates the states and transitions for a patient.

There are five distinct states: surgery (state 1), loco‐regional (local) relapse (state 2), distant relapse (state 3), and two endpoints (competing risks) which are death from breast cancer (state 4) and death from other causes (state 5). After surgery, a patient can move to four potential states, that is local relapse, distant relapse, death due to other causes, or death from breast cancer. We note that a direct transition from surgery to cancer death without having a distant relapse is highly unlikely, however it was included in the model to reflect some cases in the dataset, possibly due to not detected or annotated metastases. From local relapse, there are three possible transitions, to distant relapse, other causes, and breast cancer death. Finally, from distant relapse, there are two possible transitions, to other causes and breast cancer death. Therefore there are a total of 9 possible transitions. In cases where loco‐regional and distant relapses were detected simultaneously during the same clinical evaluation, the exact temporal sequence of appearance remains unknown. For modeling purposes, we assume a sequential progression from local (State 2) to distant (State 3). To ensure numerical stability within the transition and avoid errors associated with zero‐length intervals, a minimal increment of 0.1 months was added to the time of distant relapse in these specific instances. This approach prioritizes data integrity over arbitrary clinical assumptions regarding simultaneous occurrence frequencies, as such assumptions were not mandated by the study protocol. All patients are at risk for local relapse, distant relapse, and death after surgery. Breast cancer‐related and other causes of death are absorbing states, so there are no pathways that emerge from them. The path from one state to another is represented by an arrow, and the rate at which patients move from one state i to another j is shown by $\lambda_{\mathrm{ij}}\left(t,X_{1},\ldots ,X_{p}\right)$, where $X_{1},\ldots ,X_{p}$ are the set of covariates that have an impact in the rate.

The approach we are adopting aligns closely with the methodology proposed by [8]. When a patient enters a state, the timing is reset (clock‐reset model). Hence, time t in $\lambda_{\mathrm{ij}}\left(t\right)$ denotes the duration since transitioning into state i, not counting from the initiation of the study. Information about time spent since entering the system is included as a covariate.

Utilizing Cox's proportional hazards model, we can measure the impact that covariates have in the transition rate from state i to state j; the transition hazard rate $\lambda_{\mathrm{ij}}\left(t\right)$ at time t for this transition can be expressed in terms of the covariate vector Z representing a patient:

$$\lambda_{\mathrm{ij}}\left(t\right)=\lambda_{\mathrm{ij},0}\left(t\right)\exp\left(\beta_{\mathrm{ij}}^{T}Z\right),$$

where $\lambda_{\mathrm{ij},0}\left(t\right)$ is the baseline hazard of transition i→j, and $\beta_{\mathrm{ij}}$ is a vector of regression coefficients. Alternatively, we can write this model [8, 23] as

$$\lambda_{\mathrm{ij}}\left(t\right)=\lambda_{\mathrm{ij},0}\left(t\right)\exp\left(\beta^{T}Z_{\mathrm{ij}}\right).$$

The vector $Z_{\mathrm{ij}}$ comprises covariates that are unique to the transition from i to j and is determined based on the individual's covariates Z. Let $Z_{\mathrm{ij},k}$ represent this vector for individual k. The generalized Cox partial likelihood is as follows:

(1) $$L_{P}\left(\beta \right)=\prod_{\begin{array}{c} \text{transition}\\ i\to j \end{array}}\;\prod_{\begin{array}{c} k=1\\ d_{\mathrm{ij},k}=1 \end{array}}^{N_{i\to j}}\frac{\exp\left(\beta^{T}Z_{\mathrm{ij},k}\right)}{\sum_{l\in R_{i}\left(t_{\mathrm{ij},k}\right)}\exp\left(\beta^{T}Z_{\mathrm{ij},l}\right)},$$

where $t_{\mathrm{ij},k}$ is the time of failure or censoring for individual k corresponding to transition i→j. If $d_{\mathrm{ij},k}=1$, it indicates that individual k experienced an event for transition i→j; otherwise it is 0. Additionally, $R_{i}\left(t\right)$ denotes the risk set of state i at time t, referring to the group of individuals in state i at that specific time (t indicating the duration in state i). To simplify notation, every potential transition i→j in the process is indexed by g, g=1,…,G, where G represents the overall number of transitions possible in the process. Consequently, the partial likelihood function can be expressed as the logarithm is given by: 

(2)

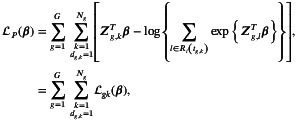


where ${ℒ}_{\mathrm{gk}}\left(\beta \right)=Z_{g,k}^{T}\beta -\log\left\{\sum_{l\in R_{i}\left(t_{g,k}\right)}\exp\left\{Z_{g,l}^{T}\beta \right\}\right\}$ is the contribution to the partial log‐likelihood of the component (g,k) (transition, patient) and $N_{g}$ denotes the number of patients in transition g.

Estimates of β and the cumulative baseline hazards $\Lambda_{g,0}\left(t\right)$, $\hat{\beta }$ and ${\hat{\Lambda }}_{g,0}\left(t\right)$ respectively, can be found by maximizing 2. The estimate of the cumulative baseline hazard of transition g is the Nelson‐Aalen estimate

$${\hat{\Lambda }}_{g,0}\left(t\right)=\sum_{\begin{array}{c} k=1\\ t_{g,k}\leq t \end{array}}^{N_{g}}\frac{d_{g,k}}{\sum_{l\in R_{i}\left(t_{g,k}\right)}\exp\left(\beta^{T}Z_{g,l}\right)}.$$

Note that the score function is

(3) $$U_{P}\left(\beta \right)=\sum_{g=1}^{G}\sum_{\begin{array}{c} k=1\\ d_{g,k}=1 \end{array}}^{N_{g}}U_{g,k}\left(\beta \right),$$

where

$$U_{g,k}\left(\beta \right)=Z_{g,k}-\left\{\sum_{l\in R_{i}\left(t_{g,k}\right)}\exp\left(\beta^{T}Z_{g,l}\right)Z_{g,l}\right\}/\sum_{l\in R_{i}\left(t_{g,k}\right)}\exp\left(\beta^{T}Z_{g,l}\right),$$

for g=1,…,G and k=1,…,N. The hessian matrix, ${\ddot{ℒ}}_{P}$, is given by

(4) $$\begin{array}{cc} {\ddot{ℒ}}_{P} & =\sum_{g=1}^{G}\sum_{\begin{array}{c} k=1\\ d_{g,k}=1 \end{array}}^{N_{g}}\frac{1}{C_{g,k}}\left\{\frac{1}{C_{g,k}}\left[\sum_{l\in R_{i}\left(t_{g,k}\right)}Q_{g,l}Z_{g,l}\right]{\left[\sum_{l\in R_{i}\left(t_{g,k}\right)}Q_{g,l}Z_{g,l}\right]}^{T}\right.\\ & \;\left.-\sum_{l\in R_{i}\left(t_{g,k}\right)}Q_{g,l}Z_{g,l}Z_{g,l}^{T}\right\}, \end{array}$$

where $Q_{g,l}=\exp\left(\beta^{T}Z_{g,l}\right)$ and $C_{g,k}=\sum_{l\in R_{i}\left(t_{g,k}\right)}Q_{g,l}$. The matrix (4) must be evaluated at $\beta =\hat{\beta }$. The large sample variance of $\hat{\beta }$ is usually approximated by $V=-{\ddot{ℒ}}_{P}^{-1}$.

## 3 Influence Diagnostics in the Multistate Model

In this section, we derive measures of influence to identify observations and/or transitions whose removal (global influence) or whose perturbation (local influence) would cause large changes in the maximum likelihood (ML) estimators.

### 3.1 Global Influence Diagnostics

The global influence technique allows studying the effect of removing observations or cases on parameter estimates. In this context, for the multistate model, only individuals in specific transitions or individuals who go through all transitions can be considered observations or cases. Transitions can not be considered cases, as a transition cannot be removed from the entire trajectory, which is a limitation of this technique for multistate models. Therefore, in this section, we consider evaluating the effect of removing a patient (case‐deletion) on the maximum likelihood estimators of β.

Following [24], we can define a measure of case‐deletion using the likelihood displacement, LD hereafter, given by

(5) $${\mathrm{LD}}_{i}\left(\beta \right)=2\left[{ℒ}_{P}\left(\hat{\beta }\right)-{ℒ}_{P}\left({\hat{\beta }}_{\left(i\right)}\right)\right],\;i=1,\ldots ,M,$$

where ${ℒ}_{P}\left(\beta \right)$ as in Equation (2), $\hat{\beta }$ and ${\hat{\beta }}_{\left(i\right)}$ are, respectively, the MLE of β considering the full data set and the data set without patient i, i=1,…,M. The expression given in (5) measures the change in the LD with estimated parameters when patient i is deleted and may be employed as global influence technique to assess the potential influence of this individual.

Following the same argument used by [25], leads to the one‐step approximation,

(6) $${\hat{\beta }}_{\left(i\right)}^{\left(1\right)}\approx \hat{\beta }-VU_{i}\left(\hat{\beta }\right),$$

where $U_{i}\left(\hat{\beta }\right)$ is the contribution of the *i*th individual to the score function, given by,

(7) $$U_{i}\left(\hat{\beta }\right)=\sum_{g=1}^{G}U_{g,i}\left(\hat{\beta }\right)I_{\mathrm{gi}},$$

with,

$$I_{\mathrm{gi}}=\left\{\begin{array}{ll} 1, & \text{if individual}\;i\;\text{was}\;a\;\text{member of the transition}\;g,\\ 0, & \text{otherwise}, \end{array}\right.$$

for g=1,…,G;i=1,…,M and $U_{g,i}\left(\hat{\beta }\right)$ as in (3) evaluated at $\beta =\hat{\beta }$.

The one‐step likelihood displacement is defined as,

(8) $${\mathrm{LD}}_{i}^{\left(1\right)}\left(\beta \right)=2\left[{ℒ}_{P}\left(\hat{\beta }\right)-{ℒ}_{P}\left({\hat{\beta }}_{\left(i\right)}^{\left(1\right)}\right)\right],\;i=1,\ldots ,M.$$

Also, using the one‐step approximation (6), the one‐step generalized Cook distance is given by, see for instance [25],

(9) $$D_{i}^{\left(1\right)}=\frac{1}{p}U_{i}^{T}\left(\hat{\beta }\right)VU_{i}\left(\hat{\beta }\right),\;i=1,\ldots ,M.$$

### 3.2 Local Influence Diagnostics

The method of local influence, based on the LD, was introduced by [11] as a general tool for assessing the sensitivity of the parameter estimates, under small changes or perturbations in the model or data set. Objective functions other than the LD have been also used for local influence analysis in [26, 27, 28]. In effect, Reference [28] extended the local influence method by replacing the LD by a more general influence measure. In our case, the likelihood equations are replaced by the estimating equations;

(10) $$\Psi \left(\hat{\beta }\right)={\left.\left\{\frac{\partial {ℒ}_{P}\left(\beta \right)}{\partial \beta }\right\}\right|}_{\beta =\hat{\beta }}=0,$$

where ${ℒ}_{P}\left(\beta \right)$ is named fit function, such as (2), that is assumed twice differentiable in β. Thus, an appropriate influence measure that generalizes the LD is defined as

$$f_{P}\left(\omega \right)=2\left[{ℒ}_{P}\left(\hat{\beta }\right)-{ℒ}_{P}\left({\hat{\beta }}_{\omega }\right)\right],$$

where $\omega ={\left(\omega_{1},\ldots ,\omega_{r}\right)}^{T}$ denotes a r‐dimensional perturbation vector.

Let Ψ(β|ω) the perturbed estimating equations by ω; and ${\hat{\beta }}_{\omega }$ the solution of $\Psi \left({\hat{\beta }}_{\omega }|\omega \right)=0$. There is a no‐perturbation vector $\omega_{0}$ such that $\Psi \left(\beta |\omega_{0}\right)=\Psi \left(\beta \right)$. The conformal normal curvature [29, 30] in the unitary direction h is given by $B_{h}=B_{h}\left(\beta \right)=\;|h^{T}\mathrm{Bh}|/\mathrm{tr}\left(B\right)$, where $B=\Delta^{T}{\left\{{\ddot{ℒ}}_{P}\right\}}^{-1}\Delta$, $0\leq B_{h}\left(\beta \right)\leq 1$, $\Delta =\partial \Psi \left(\beta |\omega \right)/\partial \omega^{T}=\partial^{2}{ℒ}_{P}\left(\beta |\omega \right)/\partial \beta \partial \omega^{T}$ evaluated at $\beta =\hat{\beta }$ and $\omega =\omega_{0}$, and ${\ddot{ℒ}}_{P}$ is the hessian matrix, defined in Equation (4). Indeed, an interesting property of the conformal curvature is that it is bounded $0\leq B_{h}\leq 1$, for any unit direction h. This normalized scale allows $B_{h}$ to be interpreted as the relative sensitivity of the model, where 0 indicates no sensitivity and 1 represents the maximum possible sensitivity in that direction.

The reader is referred to [30] for other theoretical properties of $B_{h}$, such as invariance under reparameterizations of β. The plot of the elements $|h_{\max}|$ versus i can reveal what type of perturbation has more influence on $f_{P}\left(\omega \right)$, in the neighborhood of $\omega_{0}$. Here $h_{\max}$ is the eigenvector corresponding to the largest eigenvalue of the matrix B. We denote by $B_{i}=\;|b_{\mathrm{ii}}/\mathrm{tr}\left(B\right)|$, the conformal normal curvature in the unit direction with *i*th entry 1 and all other entries 0, where $b_{\mathrm{ii}}$ form the main diagonal of the matrix B. We can use the index plot of $B_{i}$ to evaluate the presence of influential observations, the *i*th observation is potentially influential if $B_{i}>\bar{B}+2\mathrm{sd}\left(B\right)$, where $\bar{B}=\sum_{i=1}^{r}B_{i}/r$ and sd(*B*) is the standard deviation of $B_{1},\ldots ,B_{r}$.

### 3.3 Case‐Weight Perturbation Strategies

We consider in detail the case‐weight perturbation scheme, which is often the basis of the study of influence, see [11, 15]. Note that each component in (2) has the same weight in the log‐partial likelihood function of the Cox model. The purpose in this scheme of perturbation is to evaluate the sensitivity of the maximum likelihood estimators to small changes in these components. To evaluate this assumption with the local influence methodology, we consider three perturbation strategies.

- Scheme I (transition, patient): Here we are interested in pinpointing influential patients among all observations. We considered the vector of weights
$$\omega ={\left(\omega_{11},\ldots ,\omega_{1N_{1}},\omega_{21},\ldots ,\omega_{2N_{2}},\ldots ,\omega_{G1},\ldots ,\omega_{{\mathrm{GN}}_{G}}\right)}^{T},$$
  for weighting the contribution of each component in the log‐partial likelihood function. Following [31], the perturbed log‐partial likelihood function is

(11)

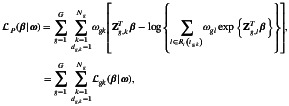

  the fit function perturbed by ω. In this case the non‐perturbation vector is $\omega_{0}=1^{T}$, a vector of dimension N×1, with $N=\sum_{g=1}^{G}N_{g}$, denotes the total number of components (transition‐individual) of the perturbed log‐partial likelihood function. Then,
$$\frac{\partial {ℒ}_{P}\left(\beta |\omega \right)}{\partial \beta }=\sum_{g=1}^{G}\;\sum_{\begin{array}{c} k=1\\ d_{g,k}=1 \end{array}}^{N_{g}}\omega_{\mathrm{gk}}\left\{Z_{g,k}-Z_{g,k}^{*}\right\},$$
  with $Z_{g,k}^{*}=\sum_{l\in R_{i}\left(t_{g,k}\right)}\omega_{\mathrm{gl}}\exp\{Z_{g,l}^{T}\beta \}Z_{g,l}/\sum_{l\in R_{i}\left(t_{g,k}\right)}\omega_{\mathrm{gl}}\exp\{Z_{g,l}^{T}\beta \}$, from where it follows that,
$$\frac{\partial Z_{g,k}^{*}}{\partial \omega_{\mathrm{gk}}}=W_{g,k}^{-1}\exp\left\{Z_{g,k}^{T}\beta \right\}\left[Z_{g,k}-Z_{g,k}^{*}\right],$$
  where $W_{g,k}=\sum_{l\in R_{i}\left(t_{g,k}\right)}\omega_{\mathrm{gl}}\exp\left\{Z_{g,l}^{T}\beta \right\}$.
  Then, the Δ matrix, of dimension p×N, takes the form,
(12) $$\Delta =\left(\Delta_{1},\ldots ,\Delta_{G}\right),\;\text{where},\;\Delta_{g}=\left(\Delta_{g,1},\ldots ,\Delta_{g,N_{g}}\right),$$
  with
$$\Delta_{g,k}=v_{\mathrm{gk}}U_{g,k}\left(\hat{\beta }\right),$$
  for g=1,…,G,
$$v_{\mathrm{gk}}=\left[1-\frac{\exp\left\{Z_{g,k}^{T}\hat{\beta }\right\}}{\sum_{l\in R_{i}\left(t_{g,k}\right)}\exp\left\{Z_{g,l}^{T}\hat{\beta }\right\}}\right],$$
  and $U_{g,k}\left(\hat{\beta }\right)$ as in (3) evaluated at $\beta =\hat{\beta }$.
- Scheme II (transition): With this scheme we are interested in identifying transitions that are outlying among all other transitions. We considered the vector of weights $\omega ={\left(\omega_{1},\ldots ,\omega_{G}\right)}^{T}$, for weighting the contribution of each possible transition of the process in the log‐partial likelihood function. In this case the non‐perturbation vector is $\omega_{0}=0^{T}$, a vector of dimension G×1. Then, the perturbed log‐partial likelihood function is
(13) $${ℒ}_{P}\left(\beta |\omega \right)=\sum_{g=1}^{G}\;\sum_{\begin{array}{c} k=1\\ d_{g,k}=1 \end{array}}^{N_{g}}\left(1+\left(N_{g}/N\right)\omega_{g}\right){ℒ}_{\mathrm{gk}}\left(\beta \right).$$
  The Δ matrix, of dimension p×G, is

(14) $$\Delta =\left(\Delta_{1\cdot },\ldots ,\Delta_{G\cdot }\right),\;\text{where},\;\Delta_{g\cdot }=\left(1/N\right)\sum_{\begin{array}{c} k=1\\ d_{g,k}=1 \end{array}}^{N_{g}}N_{g}U_{g,k}\left(\hat{\beta }\right),\;g=1,\ldots ,G.$$

iii Scheme III (patient): Without considering the transitions, we are interested in pinpointing influential patients throughout the study period. We considered the vector of weights
$$\omega ={\left(\omega_{1},\ldots ,\omega_{M}\right)}^{T},$$
  for weighting the contribution of each individual in the log‐partial likelihood function, where $M=N_{1}$ denotes the total number of patients participating in the study. In this case the perturbed log‐partial likelihood is
(15) $${ℒ}_{P}\left(\beta |\omega \right)=\sum_{g=1}^{G}\;\sum_{\begin{array}{c} k=1\\ d_{g,k}=1 \end{array}}^{N_{g}}\omega_{k}{ℒ}_{\mathrm{gk}}\left(\beta \right)=\sum_{g=1}^{G}\;\sum_{\begin{array}{c} k=1\\ d_{g,k}=1 \end{array}}^{N_{g}}{ℒ}_{\mathrm{gk}}\left(\beta |\omega \right),$$
  the fit function perturbed by ω. In this case the non‐perturbation vector is $\omega_{0}=1^{T}$, a vector of dimension M×1. Then,
$$\frac{\partial {ℒ}_{P}\left(\beta |\omega \right)}{\partial \beta }=\sum_{g=1}^{G}\;\sum_{\begin{array}{c} k=1\\ d_{g,k}=1 \end{array}}^{N_{g}}\omega_{k}U_{g,k}\left(\beta \right).$$
  Then, the Δ matrix, of dimension p×M, takes the form,
(16) $$\Delta =\left(\Delta_{1},\ldots ,\Delta_{M}\right),\;\text{where}\;\Delta_{i}=\sum_{g=1}^{G}U_{g,i}\left(\hat{\beta }\right)I_{\mathrm{gi}},$$
  with $I_{\mathrm{gi}}$ as in (7), for g=1,…,G;i=1,…,M and $U_{g,k}\left(\hat{\beta }\right)$ as in (3) evaluated at $\beta =\hat{\beta }$.

Remark 1 Using (9) and (16) we can write,

(17) $${\mathrm{pD}}_{i}^{\left(1\right)}=\Delta_{i}^{T}V\Delta_{i}=\;|b_{\mathrm{ii}}|,\;i=1,\ldots ,M.$$

Thus, in this case, the approximate generalized Cook distance is proportional to the conformal normal curvature in the direction of the *i*th patient; and therefore, the same patients will be highlighted as potentially influential by both diagnostic measures.

## 4 Results

This section presents the application of the methods presented to the METABRIC dataset [1, 22] using the model described in Section 2. Clinical management of breast cancer patients is based on the status of two biomarkers: HER2 and estrogen receptor (ER), dividing breast cancer into three main subtypes (HER2+, ER+/HER2− and ER−/HER2−) that are considered different diseases with very distinct molecular characteristics. We expect different transition functions between states for each of these subtypes. However, there is no information on HER2 status for a large proportion of the patients, so we only considered patient stratification by ER status. The multistate model shown in Figure 1 was fitted separately to ER+ and ER− patients to allow different effects and baseline transition functions for each subtype. Age was allowed to affect transitions to death from non‐malignant causes only. Tumor grade, tumor size and lymph node involvement were allowed to have different effects depending on the state of origin. The time since surgery to relapse was included as a variable in transitions from relapse [1].

Model fitting was conducted using the survival [32] and mstate [33] packages in R. Custom, R code with the implementation of the proposed local influence methodology was developed. Table B1 describes all covariables used in the fitted model and Table B2 presents the results of the fitted model.

**FIGURE 2:**
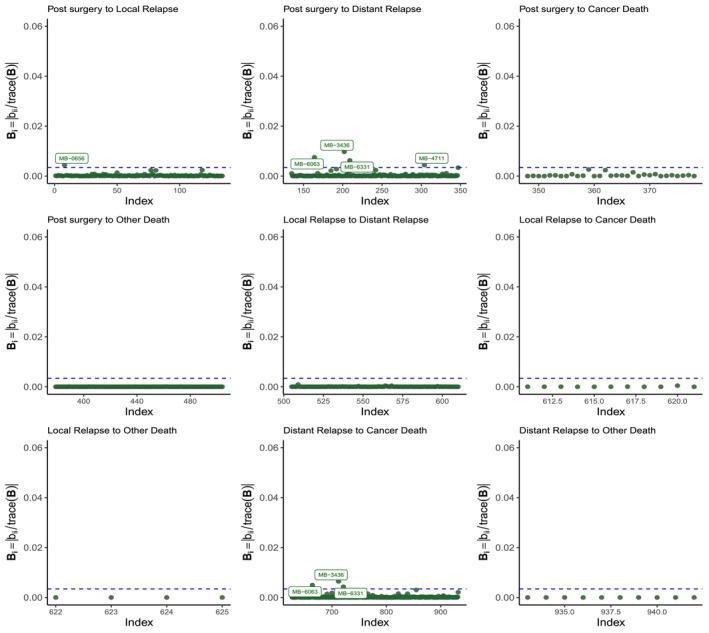
Index plots of $B_{i}$ for the Scheme I (transition, patient), for ER− patients. The dashed horizontal line indicates the cut value $\bar{B}+2\mathrm{sd}\left(B\right)$.

**FIGURE 3:**
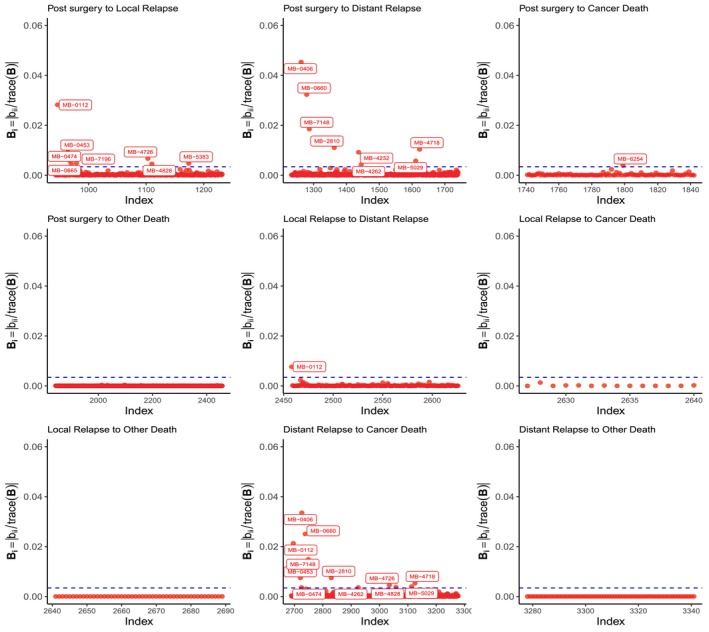
Index plots of $B_{i}$ for the Scheme I (transition, patient) for ER+ patients. The dashed horizontal line indicates the cut value $\bar{B}+2\mathrm{sd}\left(B\right)$.

After fitting the model, we performed a diagnostic analysis dividing patients according to their ER status and using the three perturbation schemes described above. For Scheme I, (transition, patient), the results are presented, separated by transition, in Figures 2 and 3. We can notice that the patients highlighted as potentially influential (with a greater impact on the likelihood function) are concentrated in the transitions post surgery to local relapse, post surgery to distant relapse and distant relapse to cancer death, with notable differences in the proportions of potentially influential patients between the ER‐negative and ER‐positive status.

**FIGURE C1:**
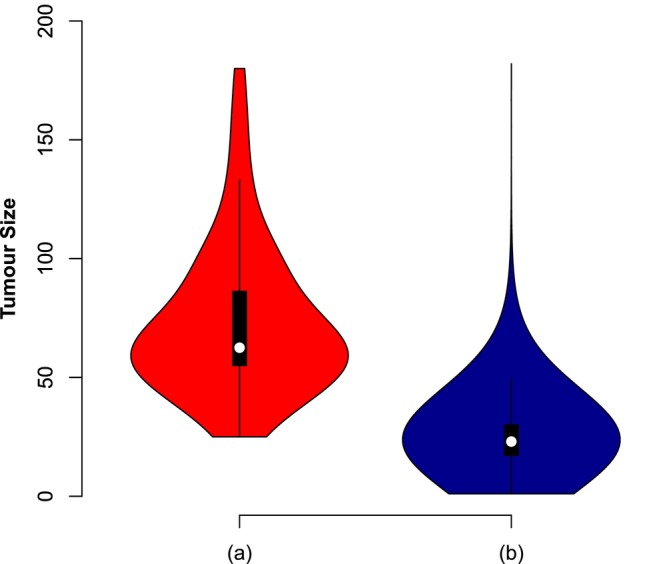
Distribution of tumor size: (a) indicates the distribution of tumor sizes among potentially influential patients, while (b) shows the tumor size distribution after removing those patients.

**FIGURE C2:**
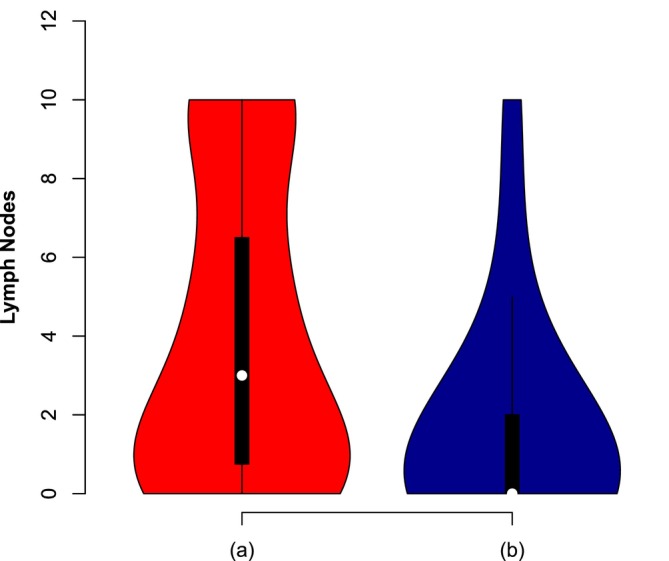
Distribution of lymph nodes: (a) indicates the distribution of lymph nodes among potentially influential patients, while (b) shows the lymph nodes distribution after removing those patients.

**FIGURE C3:**
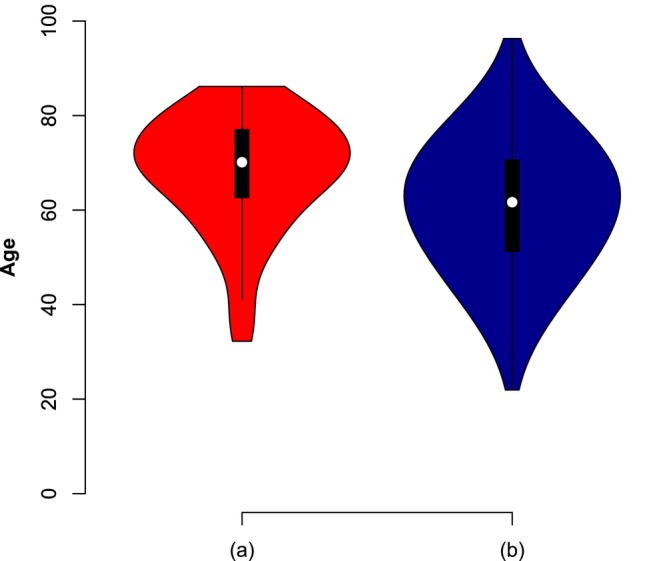
Distribution of age: (a) indicates the distribution of age among potentially influential patients, while (b) shows the age distribution after removing those patients.

For instance, in ER− cases (Figure 2), we can see that two patients stand out on these three transitions. Inspection of the values of the predictor variables indicates that both individuals had tumor sizes exceeding 100 mm, suggesting that these unusual values might have inflated the effect of size in those transitions (see Figures C1, C2, C3). They progressed very fast to distant relapse (one of them only 4 months after surgery), overestimating the hazard ratio of progressing (0.05 vs. 0.04, when these observations are removed, see Table B2), but had longer survival than expected after relapse, underestimating the hazard ratio of distant relapse to cancer death (−0.001 to 0.002).

In contrast, in the larger group of ER+ patients (Figure 3), there are several patients highlighted as potentially influential. Although these patients also presented outlier tumor sizes, their transition times were not as atypical as the patients described earlier, so the impact on the coefficients of the model is not that important, except the transitions from local relapse to distant relapse or cancer death, which changed from 0.015 to 0.025 after removal of the identified patients.

**FIGURE 4:**
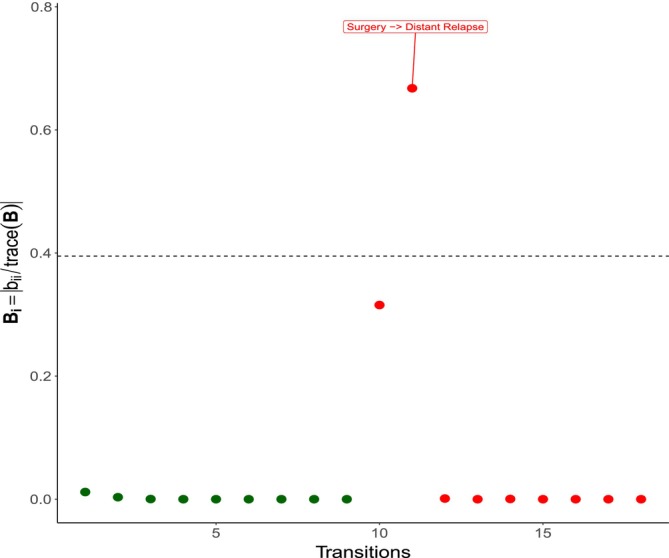
Index plots of $B_{i}$ for the Scheme II (transition). The dashed horizontal line indicates the cut value $\bar{B}+2\mathrm{sd}\left(B\right)$; dark green points indicate ER− patients, and red points indicate ER+ patients.

The results for Scheme II, (influence of transitions), are shown in Figure 4. We can observe that this perturbation scheme highlights, as potentially influential, the transitions post surgery to local relapse and post surgery to distant relapse in the ER+ patient group. Note that these two transitions provide several potentially influential patients using Scheme I. Note also that the transition distant relapse to cancer death, is not highlighted with this perturbation scheme; however, this transition brings with it several potentially influential patients using Scheme I. These influential transitions are both relapses after surgery in ER+ patients. In contrast to ER− patients, where relapses typically happen within 5 years and patients who have not relapsed by then are considered not at risk anymore, ER+ patients are characterized by a high heterogeneity in their prognosis. In particular, some of them may relapse many years after surgery, even 15–20 years. Specific molecular subgroups have been identified that may help distinguishing (to some level) individuals with a short term and a long term risk of relapse [1]. We note that our fitted model does not include any of these features (tumors are only stratified by ER status), and this heterogeneity might be the reason why those two specific transitions have a particularly high influence in the estimation of the parameters of the model.

**FIGURE 5:**
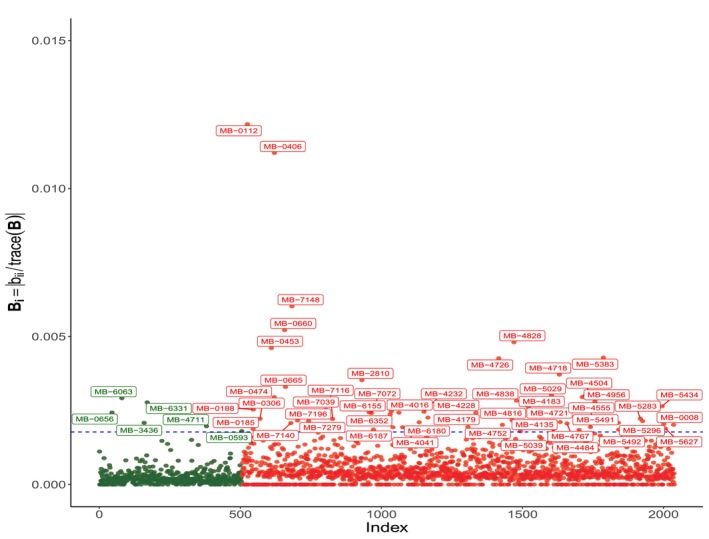
Index plots of $B_{i}$ for the Scheme III (patient). The dashed horizontal line indicates the cut value $\bar{B}+2\mathrm{sd}\left(B\right)$; dark green points indicate ER− patients, and red points indicate ER+ patients.

The Scheme III (patients) evaluates the contribution of each patient in the log‐partial likelihood function. Figure 5 highlights several potentially influential patients. Many of them have been already identified with the Scheme I. As noted, the main characteristic in these patients is a very large tumor size compared to the rest of the patients (see Figures C1, C2, C3). Those exhibiting the largest tumor sizes are primarily ER+ patients, although there are also 27 ER− patients.

**FIGURE 6:**
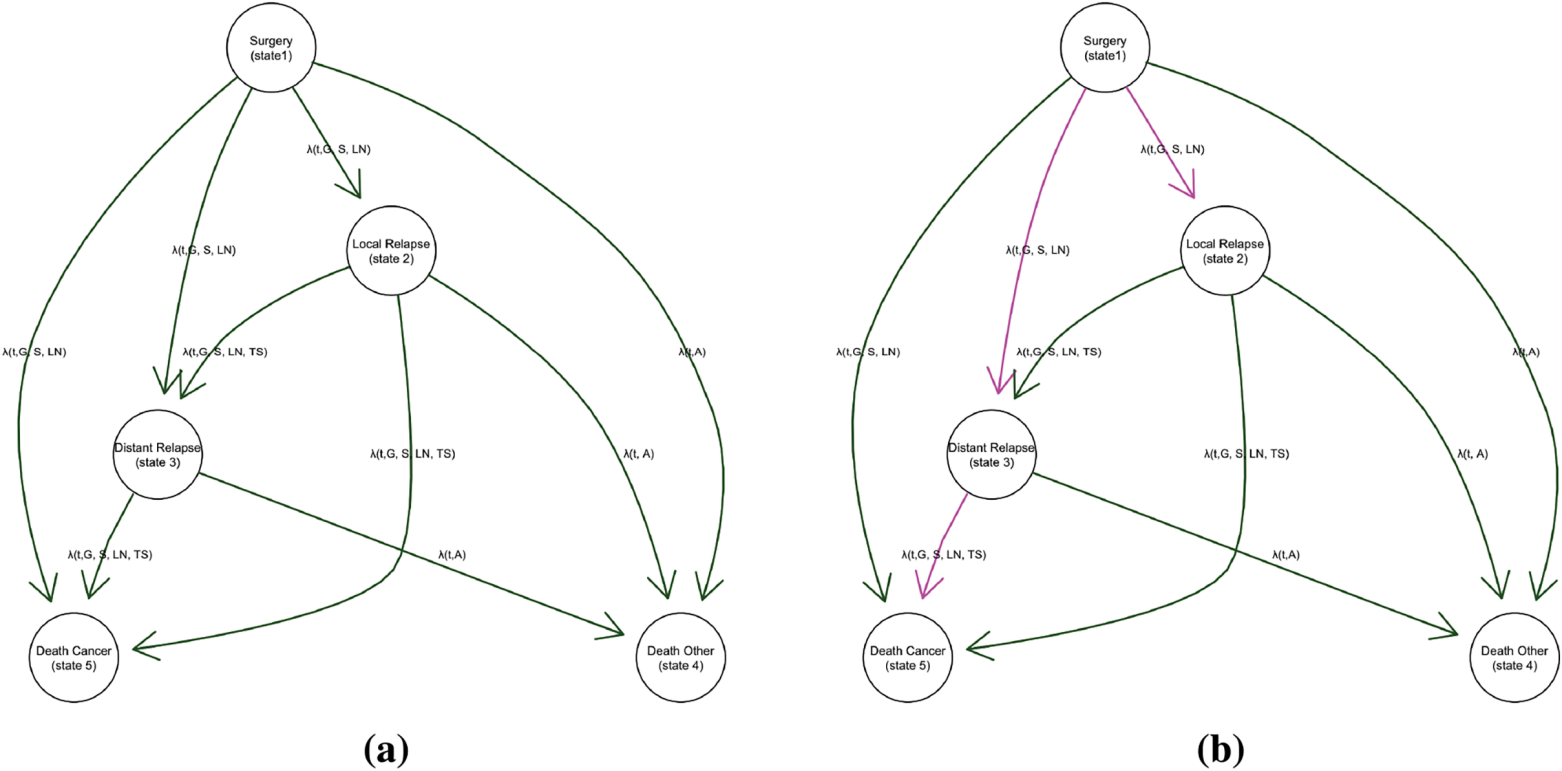
Scheme I local influence analysis. Transition diagrams: Green arcs indicate non‐influential transitions; purple arcs indicate influential transitions: (a) ER− patients and (b) ER+ patients.

In order to evaluate the impact that these patients have on the estimation of the parameters in our model, Table B2 compares the ML parameters estimated using all cases and those estimated after removing those highlighted as potentially influential under Scheme III and after removing the observations identified as influential by the Cook's distance. In this case, due to Remark 1, Equation (17), the patients highlighted by Scheme III and by Cook's distance are the same, which is reflected in the Table B2. The results in the table shows that the influence of these observations on the parameters of the model is limited to the hazard ratios of tumor size in different transitions, changing the *p*‐values in some cases from 0.109 to 0.039 in the case of transitions from local relapse in ER− patients. Figure 6 summarizes the more influential transitions for both ER− and ER+ patients.

**FIGURE D1:**
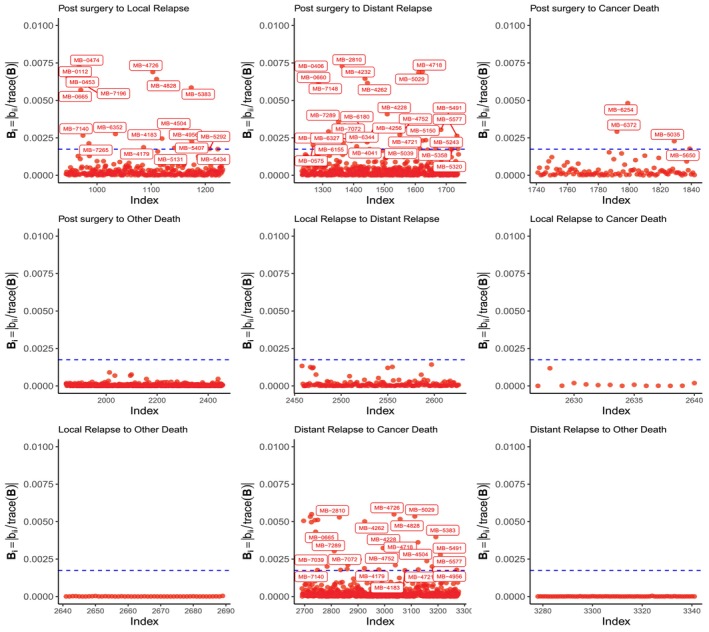
Index plots of $B_{i}$ for the Scheme I (transition, patient) for ER+ patients, modifying the covariate tumor size. All values larger than 80 mm were coded as 80 mm. The dashed horizontal line indicates the cut value $\bar{B}+2\mathrm{sd}\left(B\right)$.

**FIGURE D2:**
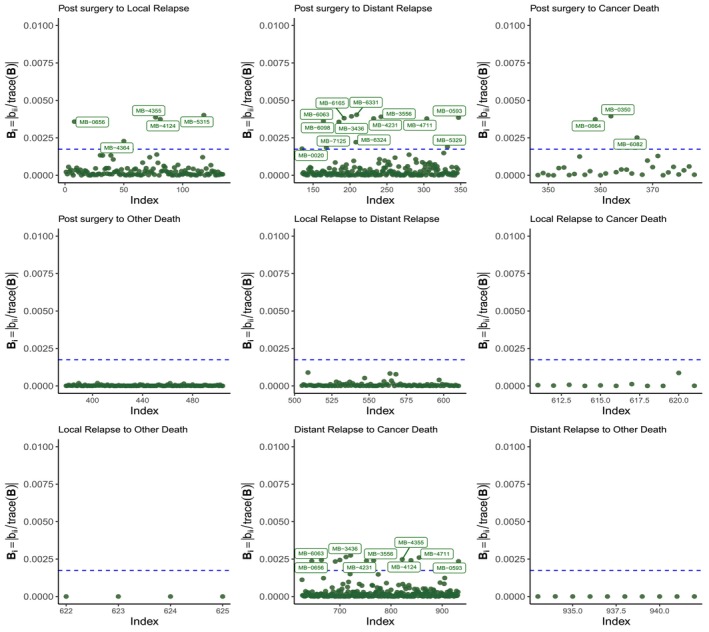
Index plots of $B_{i}$ for the Scheme I (transition, patient), for ER− patients, modifying the covariate tumor size. All values larger than 80 mm were coded as 80 mm. The dashed horizontal line indicates the cut value $\bar{B}+2\mathrm{sd}\left(B\right)$.

**FIGURE D3:**
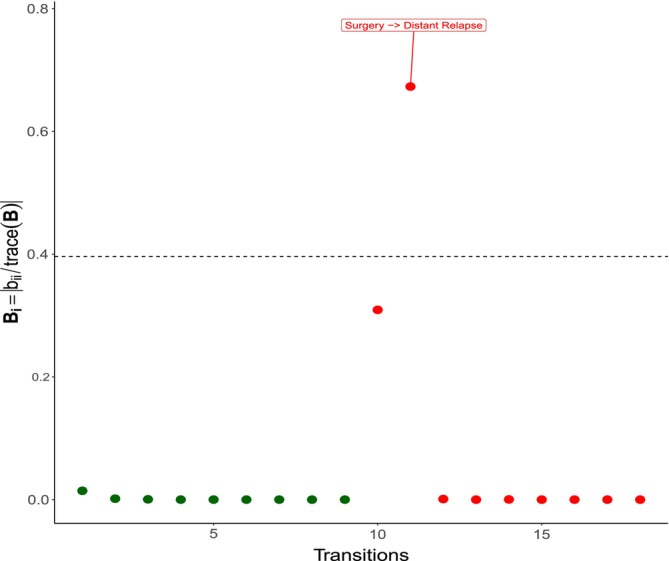
Index plots of $B_{i}$ for the Scheme II (transition) modifying the covariate tumor size. All values larger than 80 mm were coded as 80 mm. The dashed horizontal line indicates the cut value $\bar{B}+2\mathrm{sd}\left(B\right)$; dark green points indicate ER− patients, and red points indicate ER+ patients.

**FIGURE D4:**
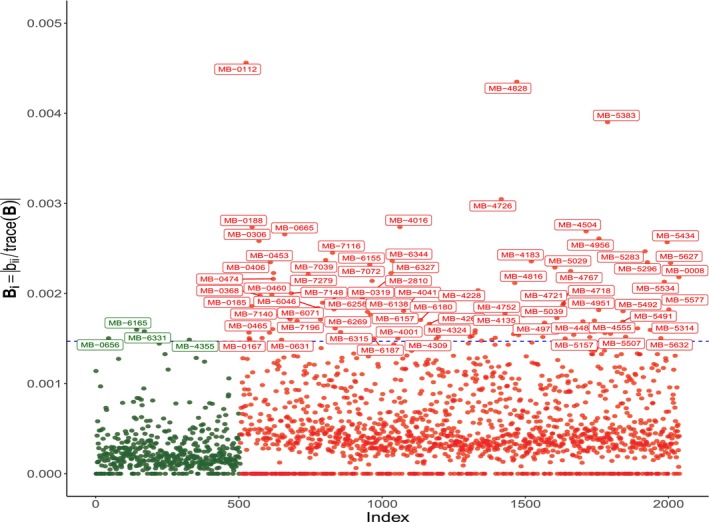
Index plots of $B_{i}$ for the Scheme III (patients) modifying the covariate tumor size. All values larger than 80 mm were coded as 80 mm. The dashed horizontal line indicates the cut value $\bar{B}+2\mathrm{sd}\left(B\right)$; dark green points indicate ER− patients, and red points indicate ER+ patients.

**FIGURE D5:**
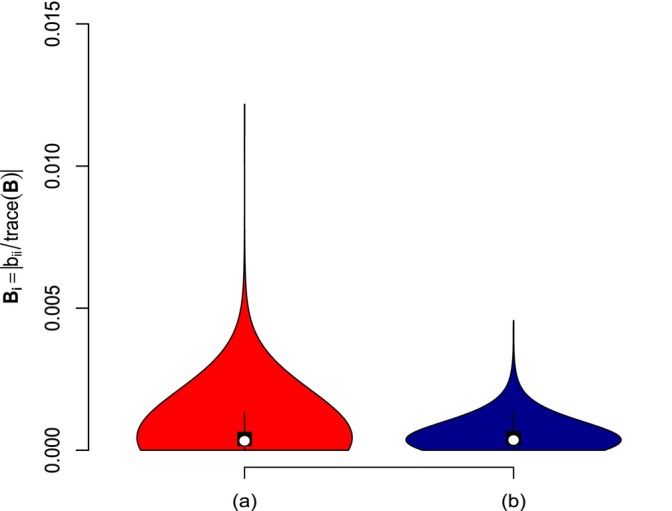
Distribution of $B_{i}$ values for the Scheme III (patients). Panel (a) displays the distribution under the original model specification, while panel (b) shows the distribution after modifying the covariate tumor size.

Motivated for this finding, we modified the variable tumor size in our model truncating large values. Specifically, all values greater than 80 mm were recoded to 80 mm to limit the impact of outliers. After refitting the model, we performed the same local influence analysis. Figures D1, D2, D3, D4 show that the values of the B coefficients are much smaller now, proving that this modified model is more robust to influential observations. See Figure D5.

We note that removing transitions for all patients under Scheme II, or for some patients under Scheme I and refitting the model for comparison can be problematic, as the new models would have a different set of parameters and we would be representing some patients more comprehensively than others.

## 5 Conclusions

In Statistics, influence analysis is a group of techniques used to evaluate the stability of statistics of interest under perturbations in the data set following the assumptions of a statistical model. This is an extremely important aspect of data analysis, as statistical models are an approximation to the natural phenomenon they describe, making essential the consideration of model adequacy [34]. Thus, the primary objective of influence diagnostics is to identify anomalous observations that could impact model adequacy and/or statistical inference.

In this work we presented a set of tools to evaluate the robustness of multistate survival models using local influence measures based on the generalized Cox partial likelihood. Closed‐form expressions were obtained for the first‐ and second‐order derivatives of the log‐partial likelihood function. We focused on case weights perturbation strategies, as it is perhaps the most widely used method to assess the influence of small modifications to the model. We derived closed‐form expressions for the perturbation matrices using a clock‐reset formulation. We note that under this alternative, the likelihood is constructed assuming that transition probabilities depend only on the elapsed time since the study onset and the current state. However, from the point of view of the local influence analysis, there is not much difference, as the likelihood function from both formulations is still derived from the Cox Proportional Hazards Model, with the minor differences of resetting the times every time a patient enters a new state.

We applied these diagnostics to our breast cancer model [1] to evaluate its robustness and identify different cases/patients/transitions that have a greater influence in the model.

The study of these three different schemes provide different views on the influence of the different pieces of the dataset. Scheme I can be interpreted as an interaction perturbation (transition, patient), while Schemes II and III can be interpreted as marginal perturbations of the transitions and patients, respectively. Furthermore, Schemes II and III treat each transition or each patient as an individual observation, assigning a unique perturbation to all observations associated to that transition/patient, and allowing to study their overall effect on the likelihood of the model. This allows, in practical studies such as our example, to evaluate the heterogeneity of specific patients with respect to their clinical pathways (Scheme III) or identify transitions that are not properly explained by the set of parameters chosen, possibly because of additional heterogeneity not included in the model, as in the case of ER+ loco‐regional and distant relapses.

Overall, we found that our model was robust, however some cases with outlier values of tumor sizes had a modest impact in the estimation on transition rates to several states. These findings would suggest a reparameterization of this variable to limit the effect of very large values. The model obtained with such reparameterization produced much smaller B values, suggesting that it would be more robust to perturbations. We note that the widely used predict [35] model uses a logarithmic transformation for this variable, which will achieve a similar effect.

Our work shows that local influence measures are a valid alternative to diagnostic measures based in case deletion (such as Cook's distance, see Remark 1) while allowing more general schemes. Future work in the multistate model can be focused on the consideration of other censoring mechanisms, such as interval censoring, or other perturbation schemes, such as covariate perturbation, as well as identifying optimal cut‐off values.

## Funding

This study was funded by a Cancer Research UK (CRUK) Career Establishment Fellowship (RCCCEA‐May22∖100002 to O.M.R.) and ANID Fondecyt de Iniciación No. 11251346. O.M.R. is further supported by UKRI grant MC_UU_00002/16.

## Conflicts of Interest

The authors declare no conflicts of interest.

## Data Availability

This study use data from the Molecular Taxonomy of Breast Cancer International Consortium (METABRIC). It can be accessed from the original publication [1]. All the functions and procedures concerning implementation included in the article have been implemented in R Core Team. The codes will be available as https://github.com/Valeria‐Leiva/Influence‐Diagnostics‐in‐Multi‐state‐Models‐for‐Breast‐Cancer/tree/main.

## Appendix A Delta Matrices for Case‐Weight Perturbation

*Scheme I*: From the Equation (11), it follows that

$$\frac{\partial {ℒ}_{P}\left(\beta |\omega \right)}{\partial \beta }=\sum_{g=1}^{G}\sum_{\begin{array}{c} k=1\\ d_{g,k}=1 \end{array}}^{N_{g}}\omega_{\mathrm{gk}}\left\{Z_{g,k}-Z_{g,k}^{*}\right\},$$

with $Z_{g,k}^{*}=\sum_{l\in R_{i}\left(t_{g,k}\right)}\omega_{\mathrm{gl}}\exp\left\{Z_{g,l}^{T}\beta \right\}Z_{g,l}/\sum_{l\in R_{i}\left(t_{g,k}\right)}\omega_{\mathrm{gl}}\exp\left\{Z_{g,l}^{T}\beta \right\}$, from where it follows that,

$$\frac{\partial Z_{g,k}^{*}}{\partial \omega_{\mathrm{gk}}}=W_{g,k}^{-1}\exp\left\{Z_{g,k}^{T}\beta \right\}\left[Z_{g,k}-Z_{g,k}^{*}\right],$$

where $W_{g,k}=\sum_{l\in R_{i}\left(t_{g,k}\right)}\omega_{\mathrm{gl}}\exp\left\{Z_{g,l}^{T}\beta \right\}$.

Then, the Δ matrix, of dimension p×N, where $N=\sum_{g=1}^{G}N_{g}$, denotes the total number of components (transition‐individual) of the perturbed log‐partial likelihood function, takes the form,

(A1)
$$\Delta =\left(\Delta_{1},\ldots ,\Delta_{G}\right),\;\text{where}\;\Delta_{g}=\left(\Delta_{g,1},\ldots ,\Delta_{g,N_{g}}\right),$$

with

$$\Delta_{g,k}=\left[1-\frac{\exp\left\{Z_{g,k}^{T}\hat{\beta }\right\}}{\sum_{l\in R_{i}\left(t_{g,k}\right)}\exp\left\{Z_{g,l}^{T}\hat{\beta }\right\}}\right]U_{g,k}\left(\hat{\beta }\right),$$

for g=1,…,G, and $U_{g,k}\left(\hat{\beta }\right)$ as in (3) evaluated at $\beta =\hat{\beta }$.

*Scheme II*: Similar to the previous case, using the Equation (13), the Δ matrix, of dimension p×G, is

(A2)
$$\Delta =\left(\Delta_{1\cdot },\ldots ,\Delta_{G\cdot }\right),\;\text{where}\;\Delta_{g\cdot }=\left(1/N\right)\sum_{\begin{array}{c} k=1\\ d_{g,k}=1 \end{array}}^{N_{g}}N_{g}U_{g,k}\left(\hat{\beta }\right),\;g=1,\ldots ,G.$$

## Appendix B Variables Used in the Analysis and Summary Statistics for the Multistate Cox Model Fit

**TABLE B1:** Description of variables used in the analysis, stratified by estrogen receptor (ER) status.

Variable	Description
ER– patients	
AGE.PS.NEG	Age at surgery in transitioning to death from other causes following surgery
AGE.LR.NEG	Age at surgery in transitioning to death from other causes following local relapse
AGE.DR.NEG	Age at surgery in transitioning to death from other causes following distant relapse
GRADE.PS.NEG	Tumor grade at surgery in transitioning to relapse or cancer death following surgery
GRADE.LR.NEG	Tumor grade at surgery in transitioning to distant relapse or cancer death following local relapse
GRADE.DR.NEG	Tumor grade at surgery in transitioning to cancer death following distant relapse
SIZE.PS.NEG	Tumor size at surgery in transitioning to relapse or cancer death following surgery
SIZE.LR.NEG	Tumor size at surgery in transitioning to distant relapse or cancer death following local relapse
SIZE.DR.NEG	Tumor size at surgery in transitioning to cancer death following distant relapse
LN.PS.NEG	Lymph nodes at surgery in transitioning to relapse or cancer death following surgery
LN.LR.NEG	Lymph nodes at surgery in transitioning to distant relapse or cancer death following local relapse
LN.DR.NEG	Lymph nodes at surgery in transitioning to cancer death following distant relapse
TLastSurgery.LR.NEG	Time since surgery in transitioning to cancer death following local relapse
TLastSurgery.DR.NEG	Time since surgery in transitioning to cancer death following distant relapse
TLastLocal.DR.NEG	Time since local relapse in transitioning to distant relapse
ER+ patients	
AGE.PS.POS	Age at surgery in transitioning to death from other causes following surgery
AGE.LR.POS	Age at surgery in transitioning to death from other causes following local relapse
AGE.DR.POS	Age at surgery in transitioning to death from other causes following distant relapse
GRADE.PS.POS	Tumor grade at surgery in transitioning to relapse or cancer death following surgery
GRADE.LR.POS	Tumor grade at surgery in transitioning to distant relapse or cancer death following local relapse
GRADE.DR.POS	Tumor grade at surgery in transitioning to cancer death following distant relapse
SIZE.PS.POS	Tumor size at surgery in transitioning to relapse or cancer death following surgery
SIZE.LR.POS	Tumor size at surgery in transitioning to distant relapse or cancer death following local relapse
SIZE.DR.POS	Tumor size at surgery in transitioning to cancer death following distant relapse
LN.PS.POS	Lymph nodes at surgery in transitioning to relapse or cancer death following surgery
LN.LR.POS	Lymph nodes at surgery in transitioning to distant relapse or cancer death following local relapse
LN.DR.POS	Lymph nodes at surgery in transitioning to cancer death following distant relapse
TLastSurgery.LR.POS	Time since surgery in transitioning to cancer death following local relapse
TLastSurgery.DR.POS	Time since surgery in transitioning to cancer death following distant relapse
TLastLocal.DR.POS	Time since local relapse in transitioning to distant relapse

*Note:* Variables describe transition frequencies between clinical states: Post‐surgery (PS), local relapse (LR), and distant relapse (DR), leading to cancer death or death from other causes.

**TABLE B2:** Summary statistics for the multistate Cox model, stratified by estrogen receptor (ER) status.

Variable	Full data		Local influence		Cook distance	
	Coef. (SE)	*p*	Coef. (SE)	*p*	Coef. (SE)	*p*
ER– patients						
AGE.PS.NEG	0.103 (0.009)	0.000	0.103 (0.009)	0.000	0.103 (0.009)	0.000
AGE.LR.NEG	0.283 (0.152)	0.062	0.283 (0.152)	0.062	0.283 (0.152)	0.062
AGE.DR.NEG	0.063 (0.032)	0.046	0.064 (0.032)	0.045	0.064 (0.032)	0.045
GRADE.PS.NEG	0.125 (0.116)	0.282	0.113 (0.117)	0.335	0.113 (0.117)	0.335
GRADE.LR.NEG	0.201 (0.245)	0.412	0.191 (0.245)	0.436	0.191 (0.245)	0.436
GRADE.DR.NEG	0.045 (0.142)	0.752	0.037 (0.144)	0.797	0.037 (0.144)	0.797
SIZE.PS.NEG	0.005 (0.003)	0.052	0.004 (0.003)	0.165	0.004 (0.003)	0.165
SIZE.LR.NEG	0.010 (0.006)	0.109	0.016 (0.008)	0.039	0.016 (0.008)	0.039
SIZE.DR.NEG	−0.001 (0.003)	0.752	0.002 (0.004)	0.718	0.002 (0.004)	0.718
LN.PS.NEG	0.124 (0.015)	0.000	0.124 (0.015)	0.000	0.124 (0.015)	0.000
LN.LR.NEG	0.003 (0.028)	0.921	−0.011 (0.030)	0.711	−0.011 (0.030)	0.711
LN.DR.NEG	0.017 (0.017)	0.308	0.020 (0.017)	0.248	0.020 (0.017)	0.248
TLastSurgery.LR.NEG	−0.111 (0.037)	0.003	−0.110 (0.037)	0.003	−0.110 (0.037)	0.003
TLastSurgery.DR.NEG	−0.076 (0.028)	0.007	−0.071 (0.028)	0.011	−0.071 (0.028)	0.011
TLastLocal.DR.NEG	0.086 (0.073)	0.237	0.080 (0.074)	0.278	0.080 (0.074)	0.278
ER+ patients						
AGE.PS.POS	0.096 (0.005)	0.000	0.096 (0.005)	0.000	0.096 (0.005)	0.000
AGE.LR.POS	0.084 (0.014)	0.000	0.085 (0.014)	0.000	0.085 (0.014)	0.000
AGE.DR.POS	0.072 (0.013)	0.000	0.073 (0.014)	0.000	0.073 (0.014)	0.000
GRADE.PS.POS	0.254 (0.055)	0.000	0.264 (0.056)	0.000	0.264 (0.056)	0.000
GRADE.LR.POS	0.115 (0.126)	0.360	0.106 (0.135)	0.431	0.106 (0.135)	0.431
GRADE.DR.POS	0.168 (0.072)	0.021	0.158 (0.077)	0.039	0.158 (0.077)	0.039
SIZE.PS.POS	0.016 (0.002)	0.000	0.014 (0.003)	0.000	0.014 (0.003)	0.000
SIZE.LR.POS	0.015 (0.004)	0.000	0.025 (0.008)	0.002	0.025 (0.008)	0.002
SIZE.DR.POS	0.000 (0.002)	0.843	0.000 (0.004)	0.962	0.000 (0.004)	0.962
LN.PS.POS	0.108 (0.012)	0.000	0.111 (0.012)	0.000	0.111 (0.012)	0.000
LN.LR.POS	0.123 (0.029)	0.000	0.154 (0.029)	0.000	0.154 (0.029)	0.000
LN.DR.POS	0.052 (0.014)	0.000	0.048 (0.015)	0.001	0.048 (0.015)	0.001
TLastSurgery.LR.POS	−0.038 (0.019)	0.050	−0.034 (0.020)	0.090	−0.034 (0.020)	0.090
TLastSurgery.DR.POS	−0.021 (0.011)	0.068	−0.016 (0.011)	0.159	−0.016 (0.011)	0.159
TLastLocal.DR.POS	0.003 (0.027)	0.904	−0.002 (0.028)	0.937	−0.002 (0.028)	0.937

*Note:* Estimated coefficients are presented with their standard errors in parentheses [e.g., 0.103 (0.009)]. For each variable, results are shown for the complete dataset, as well as for models excluding influential observations identified by local influence (Scheme III) and Cook's distance. For variable definitions, see Table B1.

**TABLE B3:** Transition frequency matrix between states for ER− patients.

From	Post‐surgery	Local relapse	Distant relapse	Cancer death	Natural death	No event	Total entering
Post‐surgery	—	140	224	37	126	323	850
Local relapse	—	—	111	12	4	13	140
Distant relapse	—	—	—	320	10	5	335
Cancer death	—	—	—	—	—	369	369
Natural death	—	—	—	—	—	140	140

**TABLE B4:** Transition frequency matrix between states for ER+ patients.

From	Post‐surgery	Local relapse	Distant relapse	Cancer death	Natural death	No event	Total entering
Post‐surgery	—	312	542	108	615	720	2297
Local relapse	—	—	176	17	49	70	312
Distant relapse	—	—	—	620	64	34	718
Cancer death	—	—	—	—	—	745	745
Natural death	—	—	—	—	—	728	728
